# Single-cell analysis reveals the COL11A1^+^ fibroblasts are cancer-specific fibroblasts that promote tumor progression

**DOI:** 10.3389/fphar.2023.1121586

**Published:** 2023-01-20

**Authors:** Jiayu Zhang, Shiqi Lu, Tong Lu, Donghui Han, Keying Zhang, Lunbiao Gan, Xinjie Wu, Yu Li, Xiaolong Zhao, Zhengxuan Li, Yajie Shen, Sijun Hu, Fa Yang, Weihong Wen, Weijun Qin

**Affiliations:** ^1^ Department of Urology, Xijing Hospital, Fourth Military Medical University, Xi’an, China; ^2^ Institute of Medical Research, Northwestern Polytechnical University, Xi’an, China

**Keywords:** cancer-associated fibroblasts, single-cell RNA sequencing, tumor microenvironment, ECM remodeling, immune response

## Abstract

**Background:** Cancer-associated fibroblasts (CAFs) promote tumor progression through extracellular matrix (ECM) remodeling and extensive communication with other cells in tumor microenvironment. However, most CAF-targeting strategies failed in clinical trials due to the heterogeneity of CAFs. Hence, we aimed to identify the cluster of tumor-promoting CAFs, elucidate their function and determine their specific membrane markers to ensure precise targeting.

**Methods:** We integrated multiple single-cell RNA sequencing (scRNA-seq) datasets across different tumors and adjacent normal tissues to identify the tumor-promoting CAF cluster. We analyzed the origin of these CAFs by pseudotime analysis, and tried to elucidate the function of these CAFs by gene regulatory network analysis and cell-cell communication analysis. We also performed cell-type deconvolution analysis to examine the association between the proportion of these CAFs and patients’ prognosis in TCGA cancer cohorts, and validated that through IHC staining in clinical tumor tissues. In addition, we analyzed the membrane molecules in different fibroblast clusters, trying to identify the membrane molecules that were specifically expressed on these CAFs.

**Results:** We found that COL11A1+ fibroblasts specifically exist in tumor tissues but not in normal tissues and named them cancer-specific fibroblasts (CSFs). We revealed that these CSFs were transformed from normal fibroblasts. CSFs represented a more activated CAF cluster and may promote tumor progression through the regulation on ECM remodeling and antitumor immune responses. High CSF proportion was associated with poor prognosis in bladder cancer (BCa) and lung adenocarcinoma (LUAD), and IHC staining of COL11A1 confirmed their specific expression in tumor stroma in clinical BCa samples. We also identified that CSFs specifically express the membrane molecules LRRC15, ITGA11, SPHK1 and FAP, which could distinguish CSFs from other fibroblasts.

**Conclusion:** We identified that CSFs is a tumor specific cluster of fibroblasts, which are in active state, may promote tumor progression through the regulation on ECM remodeling and antitumor immune responses. Membrane molecules LRRC15, ITGA11, SPHK1 and FAP could be used as therapeutic targets for CSF-targeting cancer treatment.

## 1 Introduction

Cancer-associated fibroblasts (CAFs) promote tumor invasion, metastasis and drug resistance *via* extracellular matrix (ECM) remodeling, cytokine secretion, and crosstalk with different cells in tumor microenvironment (TME), such as cancer cells, immune cells and stromal cells (Sahai et al., 2020; Biffi and Tuveson, 2021). CAFs are considered attractive targets for cancer treatment (Chen et al., 2021a). However, multiple CAF-targeting strategies failed in clinical trials and in some cases even accelerated tumor progression. For example, chemotherapy combined with CAF-targeting drugs, such as the Hedgehog pathway inhibitor hyaluronidase to decompose hyaluronic acid (hyaluronan, HA) or matrix metalloproteinase 9 (MMP9) inhibitors, did not show synergistic effects, and some combinations even increased adverse effects, such as gastrointestinal (GI) toxicity and thromboembolic (TE) events, in patients with different cancers, including metastatic pancreatic cancer (mPC), colorectal cancer (CRC), ovarian cancers (OVC) and gastric cancer (Kaye et al., 2012; Berlin et al., 2013; Catenacci et al., 2015; De Jesus-Acosta et al., 2020; Van Cutsem et al., 2020; Shah et al., 2021).

CAFs are composed of distinct clusters with different or even opposite functions, the heterogeneity of CAFs may account for the failure of these CAF-targeting treatment in clinical trials (Chen et al., 2021a; Galbo et al., 2021; Hutton et al., 2021). In addition, currently used CAF-targeting molecules are expressed in other cell types or even normal tissues, which may also explain the severe adverse effects of CAF-targeting therapeutic strategies. For example, hyaluronic acid (HA), an ECM component, also commonly exists in various human tissues, which may account for the severe adverse events of hyaluronidase (Garantziotis and Savani, 2019). The diverse origins of CAFs are an important reason for their heterogeneity, and several cell types have been proposed to be the precursors of CAFs, such as normal fibroblasts, mesenchymal stem cells (MSCs), endothelial cells, pericytes, myeloid cells and epithelial cells (Zeisberg et al., 2007; Hosaka et al., 2016; Biffi and Tuveson, 2021; Butti et al., 2021; Tang et al., 2022). In-depth analysis of the differences between these precursor cells and CAFs may provide evidence regarding the origin of CAFs and the mechanism of cell transition.

Single-cell RNA sequencing (scRNA-seq) is an effective way to analyze the heterogeneity of CAFs and the differences between different clusters of CAFs (Qian et al., 2020; Zhang et al., 2020; Olbrecht et al., 2021). The classification of CAFs is based on their different functions, by which, CAFs are commonly divided into matrix CAFs or myo-CAFs (mCAFs), inflammatory CAFs (iCAFs), antigen-presenting CAFs (apCAFs), EMT-like CAFs (eCAFs) and vascular CAFs (vCAFs) (Chen et al., 2020; Zhang et al., 2020). However, these classifications cannot distinguish the fibroblasts that specifically exist in tumor tissues from fibroblasts in normal tissues. Decoding the differences between them could not only elucidate the mechanisms how these fibroblasts promote tumor progression but also provide new therapeutic targets for cancer treatment.

In this study, by analyzing scRNA-seq datasets of multiple cancer types, we compared the differences between fibroblasts that specifically exist in tumor tissues and fibroblasts in normal tissues (Ma et al., 2019; Qian et al., 2020; Steele et al., 2020; Zhang et al., 2020; Affo et al., 2021; Chen et al., 2021b; Olbrecht et al., 2021). We identified that the COL11A1^+^ fibroblasts only exist in various tumor tissues but not in normal tissues, thus we named them cancer-specific fibroblasts (CSFs). We revealed that CSFs might transform from normal fibroblasts and may promote tumor progression through the regulation on ECM remodeling and antitumor immune responses. We also found that membrane molecules, such as leucine-rich repeat-containing protein (LRRC15), integrin alpha-11 (ITGA11), sphingosine kinase 1 (SPHK1) and fibroblast activation protein (FAP) were specifically expressed in CSFs, which could be used as therapeutic targets in CSF-targeting cancer treatment.

## 2 Materials and methods

### 2.1 Datasets of single-cell RNA-sequencing (scRNA-seq)

ScRNA-seq datasets containing tumor tissues (ten datasets across eight tumor types) and adjacent normal tissues (six datasets from five types of normal tissues) were downloaded from the Gene Expression Omnibus (GEO, RRID:SCR_005012) and https://lambrechtslab.sites.vib.be/en/data-access, as shown in Supplementary Table S1.

### 2.2 Integrated analysis of scRNA-seq datasets

Analysis of scRNA-seq datasets was performed primarily using the Seurat package (v4.0.5) in R (v4.1.0) (Hao et al., 2021). First, we used Seurat package to create individual Seurat Objects from gene expression matrices separately. Genes expressed in fewer than three cells were removed in this process. Second, strict quality control procedures were performed in Seurat. Seurat Objects were filtered to exclude the cells that expressed fewer than 200 genes, more than 6000 or 8000 genes, greater than 20% mitochondrial genes, and more than 0.1% or 1% hemoglobin genes. Third, all Seurat Objects were merged to generate a combined Seurat Object, and the merged Seurat Object was normalized and scaled separately using the NormalizeData and ScaleData functions. The Find Variable Features function was applied to identify the variable genes. Fourth, principal component analysis (PCA) was conducted with the RunPCA function based on the variable genes. After the PCA, we applied Harmony package (v0.1.0) to integrate the merged Seurat Objects and correct batch effects from different samples (Korsunsky et al., 2019). Finally, clustering was conducted using the Find Neighbors and the Find Clusters functions at a resolution = 3 for tumor tissue cells or a resolution = 0.8 for normal tissue cells. Visualization was implemented *via* uniform manifold approximation and projection (UMAP) or t-distributed stochastic neighbor embedding (tSNE).

Marker genes of cell clusters were identified using the Find All Markers function *via* Wilcoxon rank-sum tests. Then, each cell cluster was renamed to the specific cell type according to classical marker genes as follows: B cells (marked with CD79A and MS4A1), plasma cells (marked with CD79A, IGKC and IGLC2), CD4^+^ T cells (marked with CD3D, CD4 and IL7R), CD8^+^ T cells (marked with CD3D, CD8A and GZMB), dendritic cells (DCs, marked with CD1C and CD1E), endothelial cells (marked with PECAM1 and vWF), epithelial cells (marked with EPCAM and KRT18), fibroblasts (FBs, marked with COL1A1 and DCN), macrophages (marked with CD68 and CD163), mast cells (marked with CPA3 and KIT), monocytes (marked with CD14 and S100A8), natural killer cells (NK cells, marked with NKG7 and GNLY), and pericytes (marked with CSPG4 and RGS5).

### 2.3 Construction of the fibroblast atlas

The fibroblast data were extracted from integrated multi-tumor and multi-tissue scRNA-seq datasets. ScRNA-seq data of CAFs and normal fibroblasts were integrated *via* the Harmony package. Merged fibroblasts were divided into six distinct clusters. UMAP was applied to visualize the fibroblast atlas.

### 2.4 Gene set variation analysis

Gene set variation analysis (GSVA) was performed using the GSVA package (v1.40.1) (Hanzelmann et al., 2013). Specifically, single-sample gene set enrichment analysis (ssGSEA) was used to evaluate the pathway activations of the 50 hallmark gene sets from the Molecular Signatures Database (MSigDB) for each cell.

### 2.5 Gene regulatory network analysis

To identify the gene regulatory network of each fibroblast cluster, single-cell regulatory network inference and clustering (SCENIC) was performed using pySCENIC (v0.11.2), a Python implementation of the SCENIC pipeline (Van de Sande et al., 2020). First, gene expression matrix was extracted from the Seurat Object of fibroblasts using the Seurat package. Second, co-expression modules were inferred using the method of GRNBoost2 based on gene expression matrix. Third, regulons (i.e., transcription factors and their target genes) were refined from these co-expression modules using cis-regulatory motif discovery (cisTarget). Fourth, the activity of these regulons was quantified in each individual cell *via* AUCell. Finally, the differentially activated regulons of each fibroblast subcluster were identified by using the Wilcoxon rank sum test.

### 2.6 Pseudotime analysis

Pseudotime analysis was conducted using the Monocle3 package (v1.0.0) (Cao et al., 2019). Single-cell trajectories were calculated using the functions “learn_graph” and “order_cells” based on fibroblast clusters from Seurat. The result of pseudotime analysis was visualized through the UMAP method.

### 2.7 Cell–cell communication analysis

Cell–cell communication analysis was inferred based on the expression of known ligand–receptor pairs in different cell types *via* the CellChat package (v1.1.3) (Jin et al., 2021). The official workflow was used for further analysis. “Secreted Signaling” pathways were set to the reference database of ligand–receptor pairs. The essential functions “identifyOverExpressedGenes,” “identifyOverExpressedInteractions,” “projectData,” “computeCommunProb,” “computeCommunProbPathway,” and “aggregateNet” were applied using standard parameters to conduct the primary analysis. The function “netVisualbubble” was used to visualize the result of cell–cell interactions.

### 2.8 Cell composition deconvolution

We applied CIBERSORTx to perform cell composition deconvolution for TCGA bulk RNA-seq data of tumor tissues and adjacent normal tissues (Newman et al., 2019). Firstly, CIBERSORTx was used to construct a signature gene expression matrix based on the multi-cancer scRNA-seq dataset. Secondly, fragments per kilobase of transcript per million mapped reads (FPKM) values of TCGA bulk RNA-seq data were transformed into transcripts per million reads (TPM) values. Finally, cell proportions of tumor tissues and adjacent normal tissues were evaluated *via* CIBERSORTx based on the TCGA bulk RNA-seq data. The cell types included CSFs, CLDN1^+^ FBs, CXCL14^+^ FBs, BAMBI^+^ FBs, DPT^+^ FBs, RGS5^+^ FBs, B cells, CD4^+^ T cells, CD8^+^ T cells, endothelial cells, epithelial cells, macrophages, mast cells, monocytes and pericytes.

### 2.9 Immunohistochemistry (IHC) staining

IHC staining of bladder cancer tissue microarray (HBlaU079Su01, Shanghai Outdo Biotech Company) was performed with COL11A1 polyclonal antibody (1:200, ABP53753, Abbkine) according to standard protocols. Intensity of IHC staining of COL11A1 in tumor tissue was scored by two independent pathologists according to semi-quantitative immunoreactivity scoring (IRS) system. The staining intensity was scored as 0 (negative), 1 (weak), 2 (moderate), and 3 (strong), and the staining extent was quantified as: 0 (negative), 1 (1%–10%), 2 (11%–50%), 3 (51%–80%), and 4 (81%–100%). The staining intensity and extent values were multiplied to get the IHC score (Cheng et al., 2021). The baseline characteristics of enrolled bladder cancer patients showed in Supplementary Table S9.

### 2.10 Survival analysis

Survival analysis of distinct fibroblast clusters was conducted using the R packages survival (v3.2.13) and survminer (v0.4.9). Patients were divided into CSF-high and CSF-low groups in each cancer type of the TCGA cohort based on the median value of the CSF proportions. The “survfit” function was applied to generate Kaplan–Meier survival plots in different cancer types. In addition, univariable Cox proportional hazards regression analysis was performed *via* the “coxph” function. Survival analysis based on gene expression was performed *via* the Gene Expression Profiling Interactive Analysis (GEPIA, RRID:SCR_018294) platform (Tang et al., 2019).

### 2.11 Statistical analysis

Statistical analysis was conducted using R (v4.1.0). The Wilcoxon rank-rum test was performed to test the significance for most cases. Statistical significance was defined as a *p*-value <0.05 (*p < 0.05, **p < 0.01, ***p < 0.001; ns, not significant). Overall survival analysis was performed using the log-rank test.

### 2.12 Data and code availability

The scRNA-seq data analyzed in this study were obtained from https://lambrechtslab.sites.vib.be/en/data-access and the GEO (GSE141445, GSE155698, GSE125449, GSE138709, GSE142784, and GSE154170). TCGA bulk RNA-seq datasets with FPKM values and clinical data were obtained from the UCSC Xena platform (Goldman et al., 2020).

All R packages used are available online. Customized code for data analysis and plotting are available on GitHub (https://github.com/jiayu2022/pancancer_caf).

## 3 Results

### 3.1 COL11A1^+^ fibroblasts specifically exist in different tumor tissues

**FIGURE 1:**
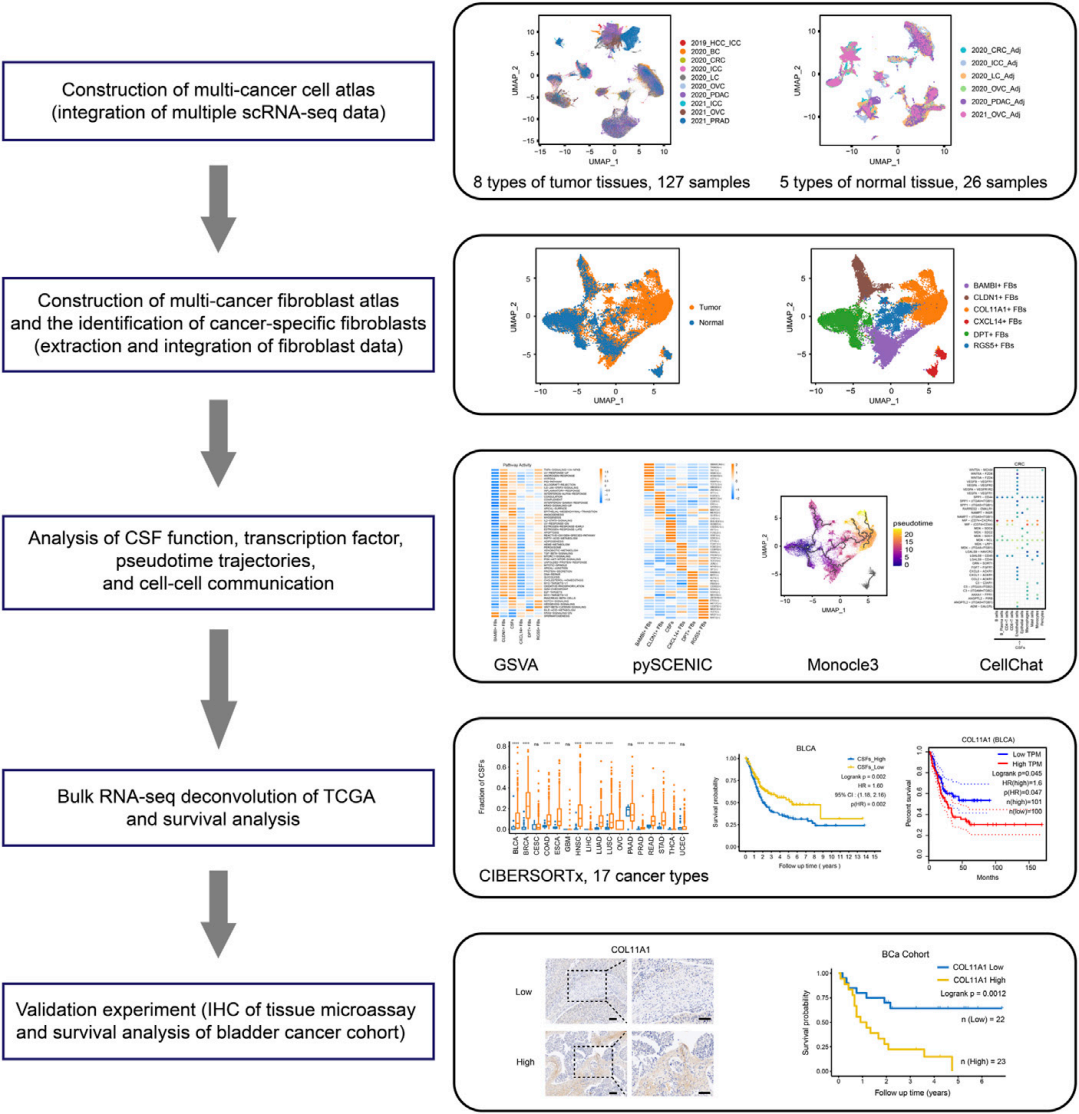
Workflow of this study.

**FIGURE 2:**
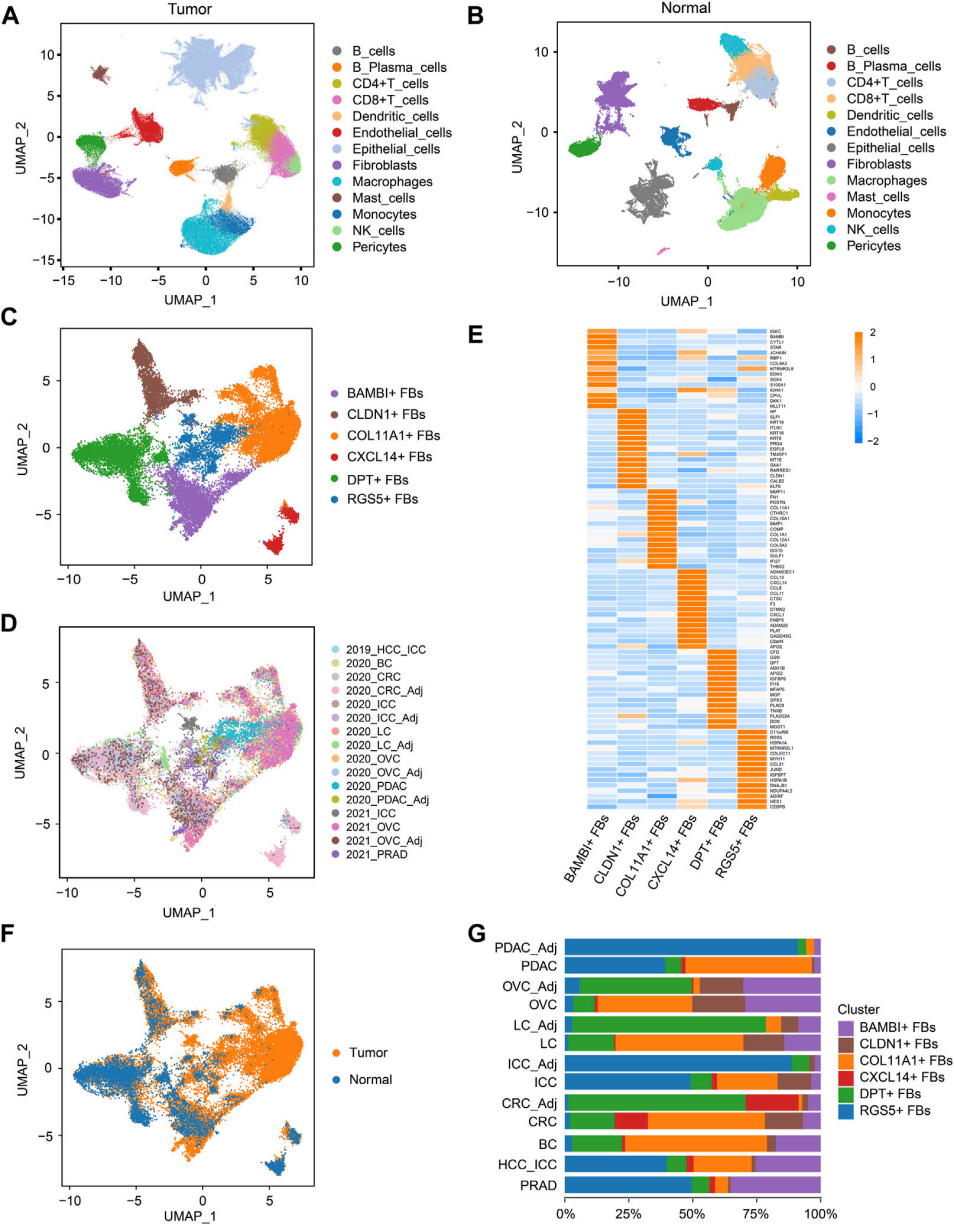
COL11A1^+^ fibroblasts specifically exist in tumor tissues. **(A)**, UMAP visualization of the cell populations in tumor tissues from ten scRNA-seq datasets across eight tumor types. **(B)**, UMAP visualization of the cell populations from six scRNA-seq datasets of five types of normal tissues. **(C)**, UMAP visualization of fibroblast clusters across normal tissues and tumor tissues. Different fibroblast clusters are color-coded. **(D)**, UMAP to depict the tissue origins of the fibroblast clusters. **(E)**, Heatmap to show the top DEGs (Wilcoxon test) in each fibroblast cluster. **(F)**, UMAP to depict the tissue types (tumor or normal tissue) of different fibroblast clusters. **(G)**, Bar plots to show the proportion of different fibroblast clusters in each tissue. DEGs, differentially expressed genes.

The graphic flowchart summarized the main procedures of present study (Figure 1). To construct a multi-tumor fibroblast atlas, we integrated ten scRNA-seq datasets across eight tumor types, including colorectal cancer (CRC), ovarian cancer (OVC), prostate adenocarcinoma (PRAD), breast cancer (BC), pancreatic ductal adenocarcinoma (PDAC), hepatocellular carcinoma (HCC), lung cancer (LC) and intrahepatic cholangiocarcinoma (ICC) (Ma et al., 2019; Qian et al., 2020; Steele et al., 2020; Zhang et al., 2020; Affo et al., 2021; Chen et al., 2021b; Olbrecht et al., 2021). The multi-tumor cell atlas included 215,871 high-quality cells from 127 tumor samples of 94 patients, and the batch effects across samples were corrected (Supplementary Figure S1A; Supplementary Tables S1, S2). These cells were divided into 13 distinct cell types using classification markers: B cells, plasma cells, CD4^+^ T cells, CD8^+^ T cells, DCs, endothelial cells, epithelial cells, fibroblasts, macrophages, mast cells, monocytes, NK cells and pericytes (Figure 2A; Supplementary Figures S1B, C; Supplementary Table S3). Similarly, six scRNA-seq datasets from five types of adjacent normal tissues were also integrated, including lung, ovary, colorectum, pancreas and intrahepatic bile duct. We obtained 65, 807 high-quality cells from 26 normal tissues, and removed the batch effects across samples (Supplementary Figure S1D; Supplementary Tables S1, S2). These cells were also divided into 13 cell types indicated above (Figure 2B; Supplementary Figure S1E, F; Supplementary Table S4).

Then, fibroblast clusters were extracted from the multi-tumor cell atlas and multi-tissue cell atlas separately, re-embedded and re-clustered to construct the multi-cancer fibroblast atlas, and the bias induced by the cell cycle states was removed (Figures 2C, D; Supplementary Figures S2A, B). In this atlas, 24,662 fibroblasts were divided into six clusters: COL11A1^+^ fibroblasts (FBs), CLDN1^+^ FBs, CXCL14^+^ FBs, BAMBI^+^ FBs, DPT^+^ FBs and RGS5^+^ FBs (Figures 2C, E; Supplementary Figures S2C, D; Supplementary Table S5). Among these clusters, CXCL14^+^ FBs mainly existed in CRC and normal colorectal tissues, indicating that they were tissue-specific fibroblasts. Notably, COL11A1^+^ FBs only existed in various tumor tissues but not normal tissues, thus, we named them cancer-specific fibroblasts (CSFs), while other clusters existed in both tumor tissues and normal tissues (Figures 2F, G). According to these results, we speculate that COL11A1^+^ FBs are CSFs that specifically exist across different cancer types.

### 3.2 CSFs represent an activated cluster of CAFs that may enhance ECM remodeling and inhibit antitumor immune response

**FIGURE 3:**
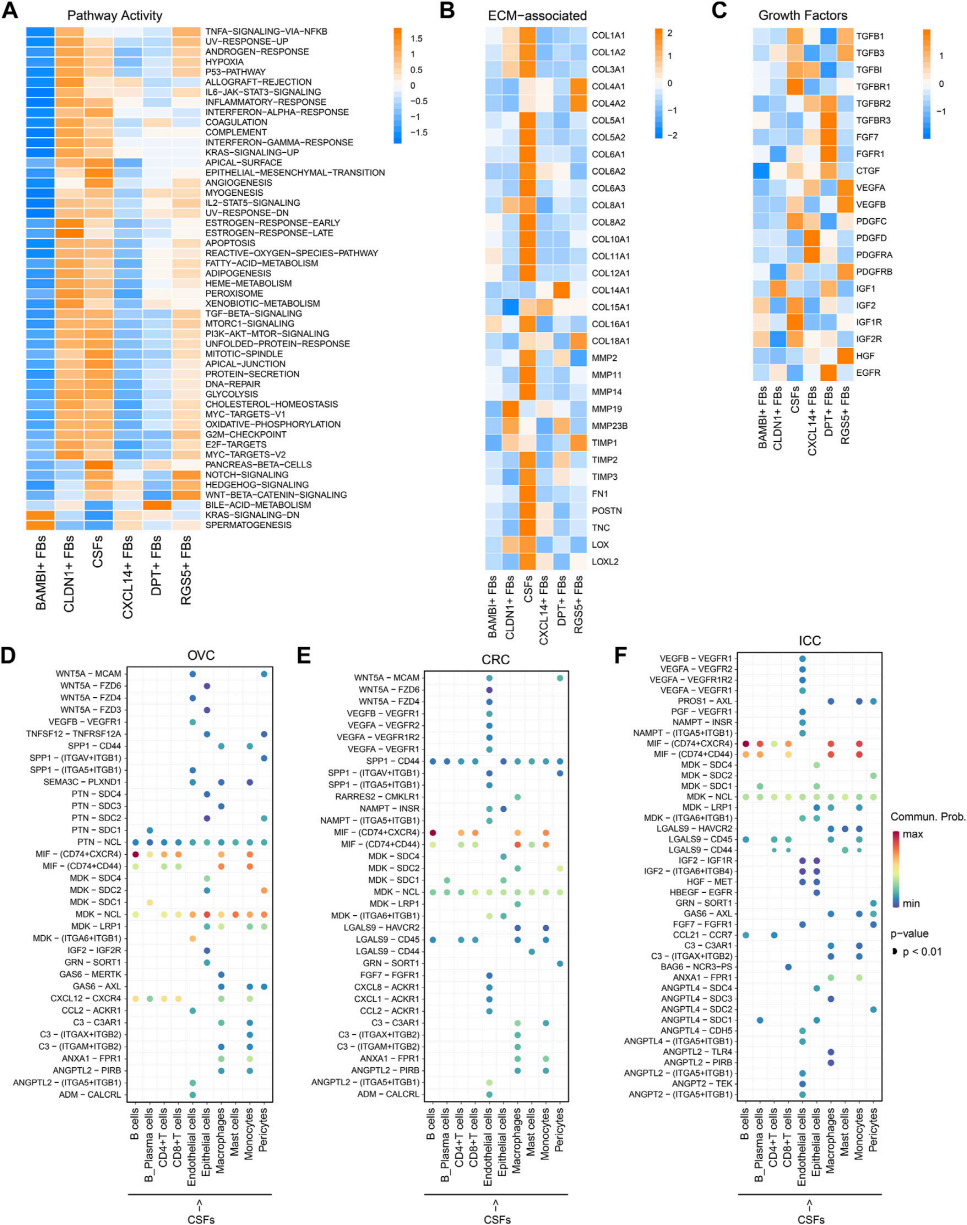
CSFs represent a more activated CAFs cluster that may enhance ECM remodeling and inhibit antitumor immune response. **(A)**, Activity of Hallmark pathways (scored per cell by GSVA) in six fibroblast clusters. **(B)**, Heatmap to show the differentially expressed ECM associated genes in six fibroblast clusters. **(C)**, Heatmap to show the differentially expressed growth factors in six fibroblast clusters. **(D–F)**, Interaction analysis to show the enriched receptor-ligand pairs between CSFs and other cell types in OVC **(D)**, CRC **(E)** and ICC **(F)**.

Next, we investigated the differences between CSFs and other fibroblast clusters. Pathway analysis revealed that CSFs had higher enrichment scores than other fibroblasts in TGF-β signaling and protein secretion pathways. Notch signaling, Hedgehog signaling and Wnt/β-catenin signaling, which have shown to be associated with the maintenance of cell stemness were highly activated in CSFs compared with other fibroblasts except the RGS5^+^ FBs (Figure 3A) (Briscoe and Therond, 2013; Liu et al., 2022; Zhou et al., 2022). Furthermore, ECM-associated genes, such as multiple collagens, MMPs, tissue inhibitors of metalloproteinases (TIMPs), fibronectin 1 (FN1) and periostin (POSTN), were all highly expressed in CSFs (Figure 3B). In addition, CSFs had high expression of TGF-β and TGF-β receptor type 1 (TGFR-1), indicating the existence of a positive feedback loop in CSFs to maintain their activation state. Similarly, the high expression of insulin-like growth factor 2 (IGF-2) and IGFR-2 indicated that IGF-2 may also promote the proliferation of CSFs in an autocrine manner (Figure 3C). These results indicate that CSFs existed in a more active state than other fibroblasts.

Then, we analyzed the potential communication between CSFs and other cell types using cell-cell communication analysis, and found that CSFs might interact with T cells and macrophages through the secretion of chemokines such as CXCL12 in OVC, LC, and PDAC (Figure 3D; Supplementary Figures S3A, B). Since CXCL12 may exert immunosuppressive function through inhibiting the infiltration of CD8^+^ T cells and promoting recruitment of regulatory T cells (Treg), myeloid-derived suppressor cells (MDSC) and macrophages (Garg et al., 2018; Givel et al., 2018; Yu et al., 2019), our findings indicated that CSFs may promote the formation of immunosuppressive microenvironment through the secretion of CXCL12. We also found that the MIF-CD74 pair was highly enriched between CSFs and immune cells, including B cells, CD4^+^ T cells, CD8^+^ T cells, macrophages and monocytes, in OVC, CRC and ICC (Figures 3D–F; Supplementary Figures S3A, B). As shown in other studies, the MIF-CD74 pair can suppress the T cell mediated antitumor effect by directly inhibiting T cell activation, or promoting the recruitment of tumor-associated macrophages (TAMs), thus accelerate tumor progression (Balogh et al., 2018; Klemke et al., 2021). Hence, CSFs may inhibit antitumor immune response *via* MIF-CD74 axis. Altogether, CSFs represent an activated cluster of CAFs, which may enhance ECM remodeling and inhibit antitumor immune response.

### 3.3 CSFs mainly transform from normal fibroblasts

**FIGURE 4:**
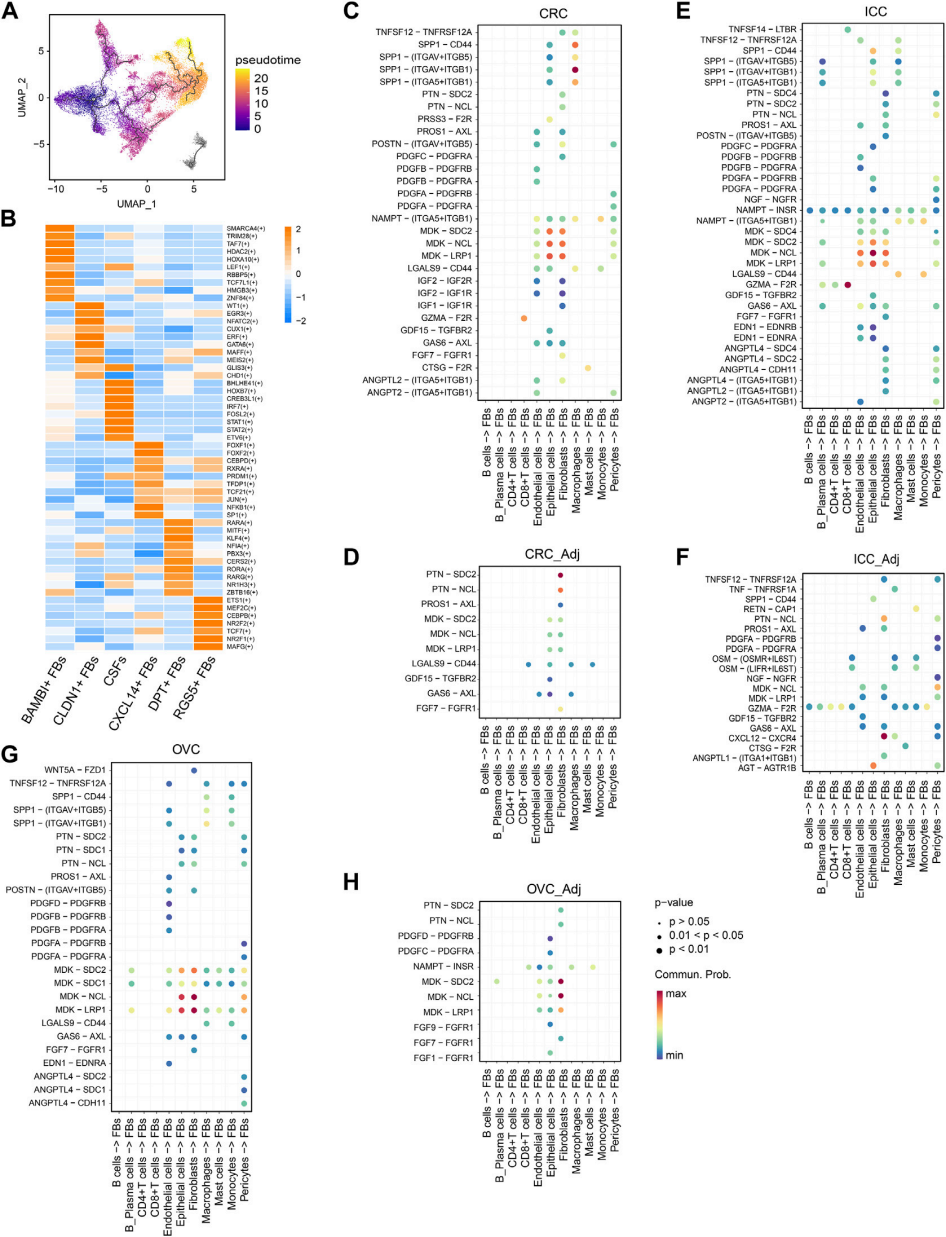
CSFs mainly transform from normal fibroblasts. **(A)**, UMAP of fibroblasts to show the projection of Pseudotime trajectory. Pseudotime values code the cell color. **(B)**, Heatmap to show the t-value for the area under the curve score of expression regulation by transcription factors, as estimated by pySCENIC. **(C–H)**, Interaction analysis to show the enriched receptor-ligand pairs between fibroblasts and other cell types in CRC **(C)**, normal colorectal tissues **(D)**, ICC **(E)**, intrahepatic bile ducts **(F)**, OVC **(G)** and normal ovarian tissues **(H)**.

To explore whether CSFs originate from other fibroblast clusters, we conducted pseudotime analysis and found that both DPT^+^ FBs and RGS5^+^ FBs could transform into CSFs. Since DPT^+^ FBs and RGS5^+^ FBs also exist in normal tissues, we speculated that CSFs transform from normal fibroblasts (Figure 4A). We also analyzed the transition of CSFs in separate cancer types, and found that in BC, CRC and OVC, CSFs were mainly transformed from DPT^+^ fibroblasts, while in ICC, CSFs were mainly transformed from RGS5^+^ FBs (Supplementary Figures S4A–D). Then, we analyzed the gene regulatory network to decode the changes in transcription factor (TF) activity during the transition. CSFs showed high activity of CREB3L1, FOSL2, IRF7 and HOXB7 (Figure 4B), among which CREB3L1, FOSL2 and IRF7 had been shown to be strongly involved in fibroblast activation, and HOXB7 could promote the transition of normal fibroblasts to MSCs (Steens et al., 2020; Tabib et al., 2021). These results indicate that CREB3L1, FOSL2, IRF7 and HOXB7 may facilitate the transition of normal fibroblasts to CSFs.

Then, we compared the differences in secreted proteins between tumor tissues and normal tissues to identify the factors that might be responsible for the activation and transition of CSFs. We found that the osteopontin (OPN, encoded by SPP1)-CD44 pair was significantly enriched between macrophages and fibroblasts in different tumor types but was absent in most normal tissues (Figures 4C–H; Supplementary Figures S5A–D). As reported in other studies, OPN/SPP1 could indeed induce the transition of normal fibroblasts into tumor-promoting CAFs (Sharon et al., 2015; Butti et al., 2021). However, in contrast to other reports that showed OPN/SPP1 was highly expressed in tumor cells, our data indicated that OPN/SPP1 was mainly expressed in macrophages (Supplementary Figure S5E). Since it has been well recognized that macrophages are critical for the transition of normal fibroblasts into CAFs (Costa-Silva et al., 2015; Nielsen et al., 2016), our results indicate that macrophages may promote the transition of normal fibroblasts to CSFs through OPN/SPP1-CD44 axis.

### 3.4 High CSF proportion is associated with poor prognosis in bladder cancer and lung adenocarcinoma

**FIGURE 5:**
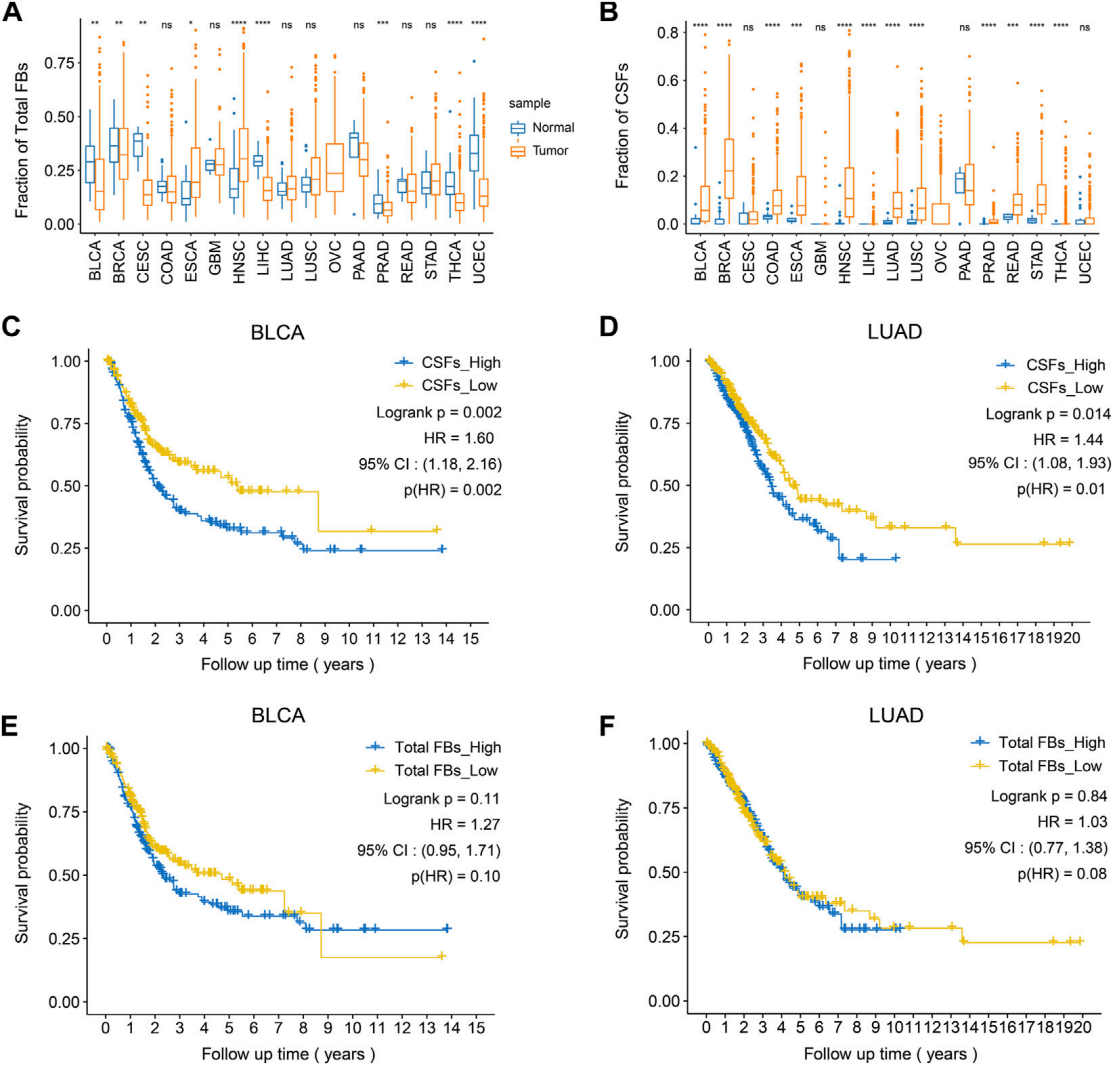
High CSFs proportion is associated with poor prognosis in bladder cancer and lung adenocarcinoma. **(A)**, The fractions of total fibroblasts in tumor tissues and normal tissues in 17 TCGA cancer types. **(B)**, The fractions of CSFs in tumor tissues and normal tissues in 17 TCGA cancer types. **(C, D)**, Kaplan-Meier plots to depict the survival of patients with high CSFs or low CSFs in BLCA **(C)** and LUAD **(D)**. **(E, F)**, Kaplan-Meier plots to depict the survival of patients with high or low fibroblasts in BLCA **(E)** and LUAD **(F)**. HR, hazard ratio.

After confirming the existence of CSFs in different tumors, we explored the association between CSF proportion and patients’ prognosis. For this purpose, we assessed cell proportions of tumor tissues and adjacent normal tissues in multiple cancer cohorts from TCGA database. CIBERSORTx was used for cell composition deconvolution analysis, with the scRNA-seq dataset of multi-cancer as a reference panel (Supplementary Table S8). Results showed that the proportions of total fibroblasts in most tumor types were similar to or even lower than adjacent normal tissues, which might be caused by the increased proportions of epithelial cells in tumor tissues (Figure 5A). However, the proportions of CSFs were significantly higher in most types of tumor tissues (12 out of 17) than adjacent normal tissues, indicating that CSFs mainly exist in tumor tissues (Figure 5B). Consistently, the expression of COL11A1 in various tumor tissues was significantly higher than adjacent normal tissues (Supplementary Figure S6).

Then, we examined the association between CSF proportion and patients’ prognosis, in which patients with different cancer types were divided into two groups based on the proportion of CSFs (high or low) for survival analysis (Supplementary Figure S7). As a control, we also examined whether total fibroblast proportion was associated with patients’ prognosis. Results showed that high CSFs proportion was associated with poor prognosis in bladder cancer (BCa) and lung adenocarcinoma (LUAD), while total fibroblast proportion was not associated with clinical outcomes, indicating that CSFs may promote tumor progression in BCa and LUAD (Figures 5C–F).

### 3.5 Highly expressed ECM-associated genes in CSFs are also associated with patients’ prognosis

**FIGURE 6:**
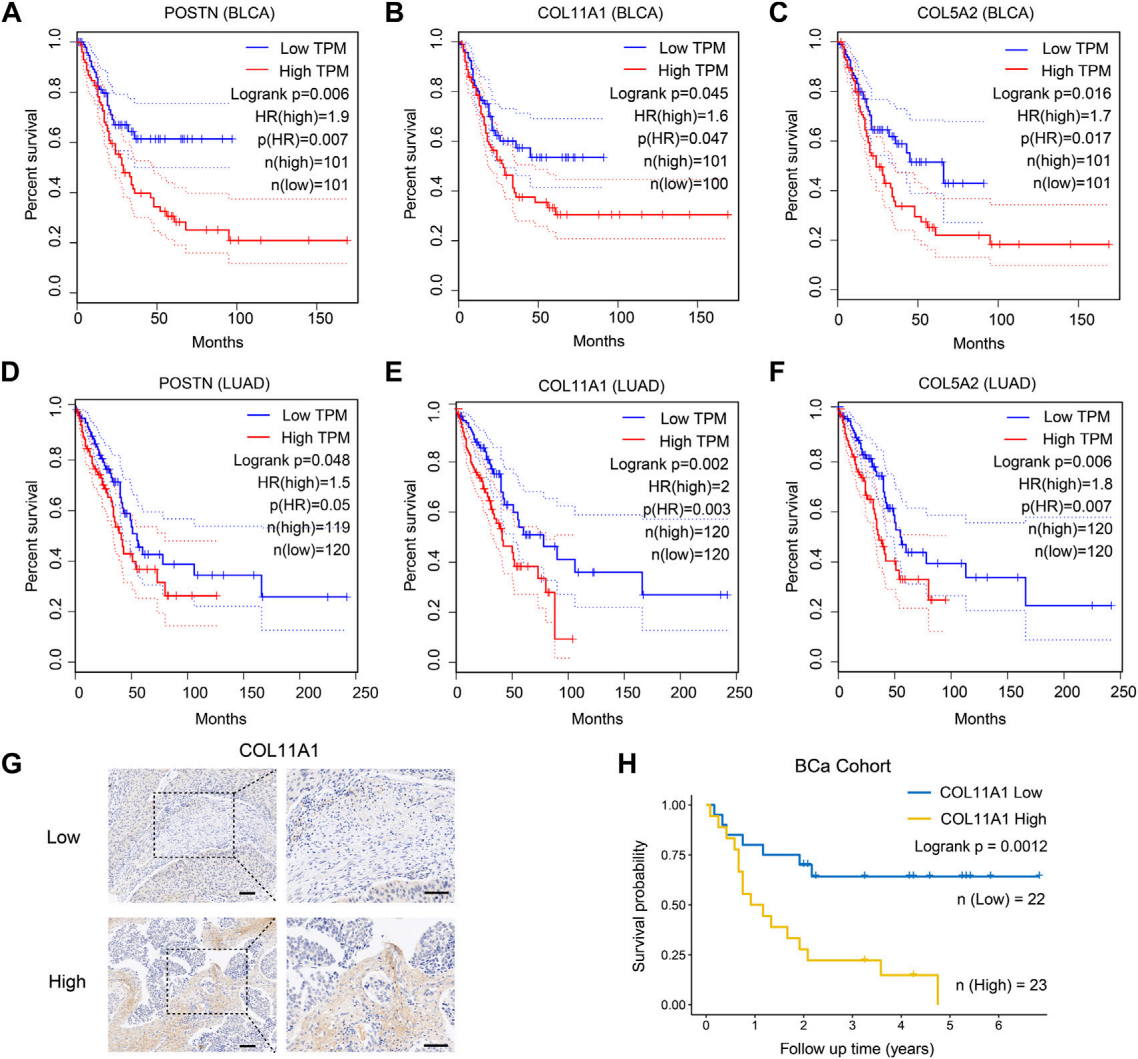
ECM associated genes are highly expressed in CSFs and associated with patients’ prognosis. **(A–C)**, Overall survival of patients with different expression of the three highly expressed genes in CSFs, including POSTN **(A)**, COL11A1 **(B)** and COL5A2 **(C)** in TCGA BLCA cohort. **(D–F)**, Overall survival of patients with different expression of the three highly expressed genes in CSFs, including POSTN **(D)**, COL11A1 **(E)** and COL5A2 **(F)** in TCGA LUAD cohort. **(G)**, IHC analysis of COL11A1 expression in BCa tissues. Scale bar, 50 μm. **(H)**, Overall survival of patients with different COL11A1 expression in BCa cohort (IHC score ≤1, low expression of COL11A1; IHC score >1, high expression of COL11A1).

As previously shown in Figure 3B, compared with other clusters of fibroblasts, CSFs express higher levels of multiple collagens, MMPs, TIMPs, FN1 and POSTN, indicating that CSFs might be involved in ECM remodeling. To evaluate whether CSFs promote tumor progression through ECM remodeling, we studied the association between the highly expressed ECM associated genes in CSFs and patients’ prognosis in BCa and LUAD cohorts from TCGA database. Results showed that ECM associated genes, such as POSTN, COL11A1 and COL5A2, were also associated with poor prognosis in BCa and LUAD patients (Figures 6A–F). Furthermore, we performed IHC staining to examine the expression of COL11A1 in clinical BCa samples and verified the specific COL11A1 expression in tumor stroma. We validated that patients with high COL11A1 expression tended to have poor prognosis in our BCa cohort (Figures 6G, H). We also confirmed that these ECM associated genes were mainly expressed in fibroblasts, especially CSFs (Supplementary Figure S8). Our data was consistent with other studies which also showed that POSTN and COL11A1 were more highly expressed in tumor tissues than normal tissues (Raglow and Thomas, 2015; Yu et al., 2018), POSTN could promote cancer stemness in ovarian cancer and head and neck squamous cell carcinoma (HNSCC) (Malanchi et al., 2011; Yu et al., 2018), and COL11A1 could facilitate fibroblast activation through modulating the TGF-β pathway and contribute to metastasis and poor clinical outcomes in ovarian cancer (Cheon et al., 2014; Wu et al., 2021). Altogether, these results further confirmed that CSFs may promote tumor progression through enhancing ECM remodeling.

### 3.6 CSFs specifically express membrane proteins FAP, LRRC15, ITGA11 and SPHK1

**FIGURE 7:**
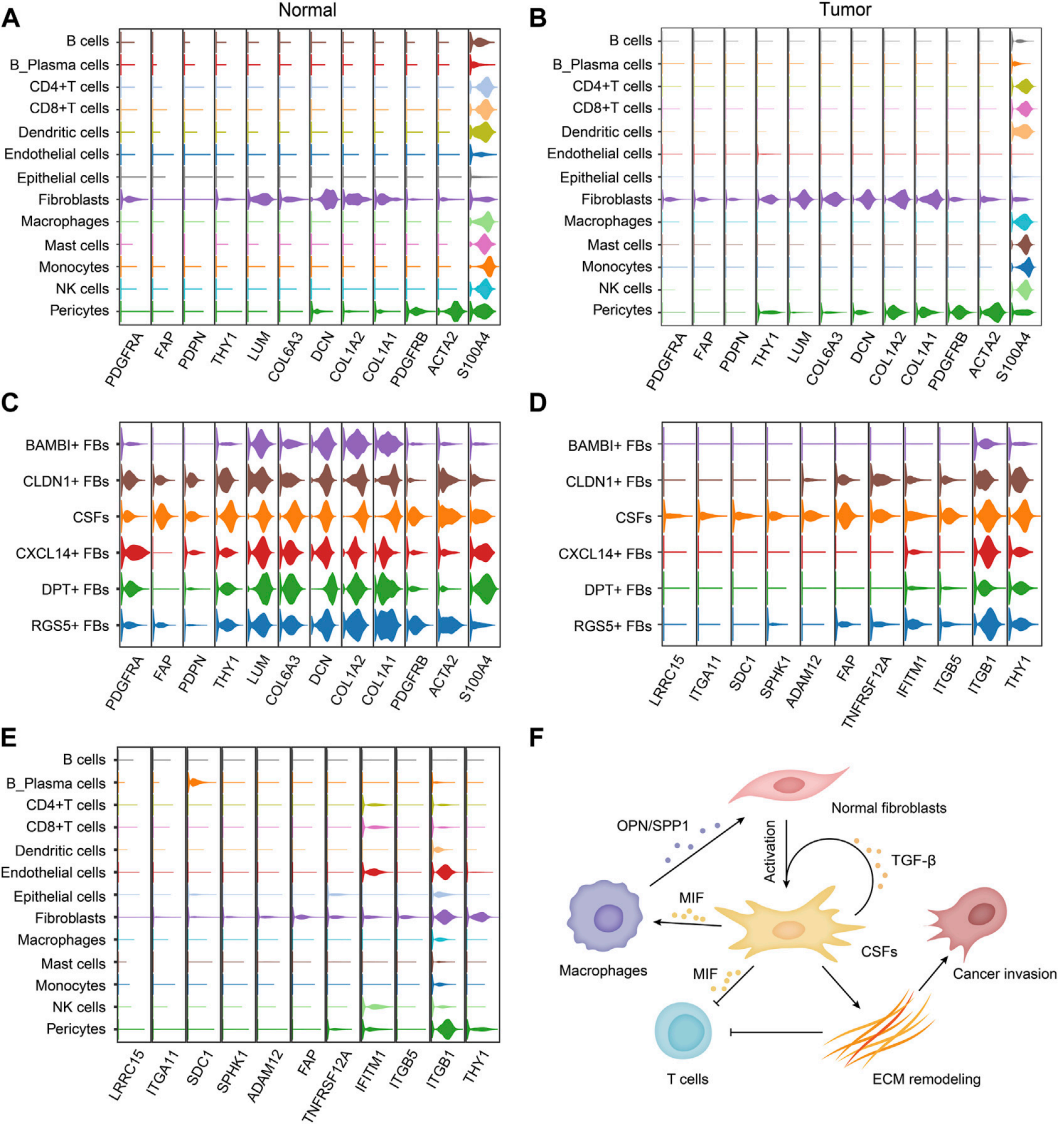
CSFs specifically express membrane proteins LRRC15, ITGA11, SPHK1 and FAP. **(A, B)**, Violin plots to show the expression levels of classical fibroblast markers in different cell types of normal tissues **(A)** and tumor tissues **(B)**. **(C)**, Violin plots to show the expression levels of classical fibroblast markers in different fibroblast clusters. **(D, E)**, Violin plots to show the highly expressed surface molecules of CSFs in different fibroblast clusters **(D)** and different cell types **(E)**. **(F)**, Schematic illustration of the proposed mechanism how CSFs promote tumor progression.

Because of the tumor-promoting function, CSFs can be potential targets for cancer treatment. Therefore, identifying the membrane molecules that are specifically expressed on CSFs is critical for CSFs specific targeting. We first examined the expression of classical fibroblast marker genes in different fibroblast clusters. Platelet-derived growth factor receptor alpha (PDGFR-α, encoded by PDGFRA) was specifically expressed in fibroblasts but not in other cell types, but since it was expressed in fibroblasts of both normal tissues and tumor tissues, thus it could be used as a pan-fibroblast marker (Figures 7A, B). We found that, α-smooth muscle actin (α-SMA, encoded by ACTA2), a commonly used marker for CAFs, was not a specific marker for CAFs since it was also highly expressed in pericytes, which was consistent with other previously reported studies (Schadler et al., 2010; Dubrac et al., 2018). In contrast, we found that FAP and PDPN were specifically expressed in fibroblasts in tumor tissues but almost absent in normal tissues, indicating that they could be used as CAFs specific markers in various cancer types (Figures 7A, B). In addition, we found that FAP was mainly expressed in CSFs, very few in other clusters, indicating that it could be an attractive CSFs specific target (Figure 7C).

To find out more CSFs specific membrane molecules for targeting, we also screened the membrane molecules in different fibroblast clusters. We found that LRRC15, ITGA11 and SPHK1 were also specifically expressed on CSFs, but not in other clusters of fibroblasts, indicating that they could also be attractive CSFs specific targets for cancer treatment (Figures 7D, E). Thus, we proposed that FAP, LRRC15, ITGA11 and SPHK1 could be used as markers for CSFs targeting, especially LRRC15 and ITGA11 due to their more specific expression.

Based on all the results, we proposed a working model how CSFs promote tumor progression, that is, CSFs secrete plenty of TGF-β to maintain their activation state and enhance ECM remodeling; CSFs also highly express MIF to inhibit T cell-mediated antitumor immune response; MIF may also induce macrophage to secrete OPN/SPP1, thus further enhance the transition of normal fibroblasts to CSFs (Figure 7F).

## 4 Discussion

CAFs have long been considered attractive targets for cancer treatment since they can promote tumor progression through ECM remodeling and extensive interactions with other cell types (Sahai et al., 2020; Yang et al., 2020; Chen et al., 2021a; Biffi and Tuveson, 2021). However, clinical trials targeting CAFs have not met the expectations, which might be caused by the heterogeneity of CAFs, since they are composed of distinct clusters that have different even opposite functions (Kim et al., 2014; Yang et al., 2020; Chen et al., 2021a; Biffi and Tuveson, 2021; Chen et al., 2021c; Hutton et al., 2021). Thus, to improve the antitumor efficacy of CAFs targeting strategies, it is of vital important to identify the tumor-promoting CAF clusters and elucidate their function in tumor progression.

ScRNA-seq provides an effective way to study the heterogeneity of CAFs and decode the differences between different CAF clusters (Zhang et al., 2020; Galbo et al., 2021; Olbrecht et al., 2021). Currently, based on scRNA-seq data, CAFs are commonly divided into mCAFs, iCAFs, apCAFs, vCAFs and eCAFs (Chen et al., 2020; Zhang et al., 2020). In addition, some new markers have been identified to classify CAFs into other clusters. For example, Hutton et al. found that in pancreatic cancer, CD105^+^ and CD105‾ fibroblasts had opposite functions; CD105^+^ fibroblasts could promote tumor progression, while CD105‾ fibroblasts inhibited tumor growth by enhancing the antitumor immune response. Thus, in their study, CAFs were divided into tumor-promoting CAFs and tumor-suppressing CAFs. In a study of non-small-cell lung cancer (NSCLC), based on their response to tyrosine kinase inhibitors (TKIs), Hu et al. divided CAFs into three clusters with distinctive function (Hu et al., 2021). Although these studies proposed different mechanisms how CAFs promote tumor progression, they did not clarify the differences between fibroblasts that specifically exist in tumor tissues (we called CSFs) and normal fibroblasts. Therefore, it was still difficult to identify therapeutic targets for CSFs. In addition, current studies were mainly performed in mouse models, however, CAF clusters are more complex in human tumors than mouse models. Thus, it’s still urgently needed to identify the CSF cluster in human sample.

In this study, by using scRNA-seq data from multiple cancer types, we compared the differences between fibroblasts specifically exist in tumor tissues and normal tissues. We constructed a multi-cancer fibroblast atlas, in which fibroblasts were classified into six clusters: BAMBI^+^ FBs, CLDN1^+^ FBs, COL11A1^+^ FBs, CXCL14^+^ FBs, DPT^+^ FBs and RGS5^+^ FBs. Among these clusters, BAMBI^+^ FBs have high expression of genes that are related to Wnt signaling pathway, such as BAMBI, SOX4, DKK1 and MDK. CLDN1^+^ FBs highly express keratins such as KRT8, KRT18 and KRT19, indicating that they are epithelial-to-mesenchymal transition (EMT)-like fibroblasts. CXCL14^+^ FBs have high expression of chemokines, including CCL8, CCL11, CCL13, CXCL1 and CXCL14, indicating that they are inflammatory fibroblasts. DPT^+^ FBs have high expression of APOD, DPT, CFD, GSN and MGP, indicating that they are associated with lipid metabolism. RGS5^+^ FBs have high expression of genes related with vascular development, including RGS5, MYH11 and NOTCH3. Notably, COL11A1^+^ FBs only existed in various tumor tissues but not in normal tissues, while other fibroblast clusters existed in both tumor tissues and normal tissues. Thus, we mainly focused on the COL11A1^+^ FBs and named them CSFs.

Interestingly, one previous study has shown that coordinated overexpression of COL11A1, THBS2 and INHBA in a subset of CAFs was related to high-stage cancers, and this signature only occurred when cancers reached certain stages, for example stage IIIc in ovarian cancer and stage II in colorectal cancer. This subset of CAFs was named metastasis associated fibroblasts (MAFs) (Kim et al., 2010). Then by comparing the gene expression profile at single cell level, the same group further reported that these COL11A1-expressing CAFs were transformed from a particular type of adipose derived stromal/stem cells (ASCs) that also naturally present in the stromal vascular fraction of normal adipose tissue (Zhu et al., 2021). Since ASCs are also kind of normal fibroblasts, since they express fibroblast marker genes, our findings are consistent that CSFs originate from normal fibroblasts. It’s more interesting that when analyzing the origin of CSFs in separate cancer types, we found that CSFs were mainly transformed from DPT^+^ FBs in BC, CRC and OVC, while mainly from RGS5^+^ FBs in ICC. These results indicated that CSFs may originate from different normal fibroblast clusters in different cancer types.

Some other studies reported that COL11A1 was also involved in the CAF-cancer cell interaction and promote tumor progression through different mechanisms. For example, one study analyzed three large microarray datasets in serous ovarian cancer, and reported a 10-gene signature that are associated with poor OS, which included COL11A1 and could be regulated by TGF-signaling. They also found that COL11A1 expression increased during ovarian cancer progression, and downregulation of COL11A1 in ovarian cancer cells could significantly inhibit tumor growth *in vivo* (Cheon et al., 2014). The same group also found that COL11A1 could be used as a specific marker for activated CAFs, and COL11A1 expression was correlated with tumor stage, tumor grade and patients’ outcome in 13 types of carcinomas (Jia et al., 2016). In addition, another study reported that COL11A1 could induce the expression and secretion of TGF-β3 in ovarian cancer cells through NFκB/IGFBP2 axis, which then promote the transformation of ovarian fibroblasts into CAFs, at the same time, COL11A1 could also induce CAFs to secrete IL-6, thus to promote ovarian cancer cell growth and invasion (Wu et al., 2021). Based on these findings that COL11A1 could promote tumor progression, COL11A1 has been considered as a potential therapeutic target for cancer treatment (Liu et al., 2021). Although most studies showed that COL11A1 was mainly expressed in CAFs, yet COL11A1 was also found to be expressed in certain kind of tumor cells, such as the tumor cells of salivary gland cancer (SGC) with intercalated duct origin. This finding indicated that COL11A1-targeted therapy might have particularly high potential in SGC, or could help to categorize tumors in the setting of possible future COL11A1-related therapies (Arolt et al., 2022).

In our study, we aimed to identify the fibroblasts that specifically exist in tumor tissues. Our results showed that COL11A1^+^ FBs specifically exist in tumor tissues, thus could be considered as CSFs. Then we further analyzed their origin and the potential mechanisms how CSFs promote tumor progression. By using pseudotime analysis, we revealed that these CSFs might transformed from normal fibroblasts. By using pathway analysis, gene regulatory network analysis and cell-cell communication analysis, we found that CSFs exhibited a higher activation state than other fibroblast clusters. The high expression of ECM-associated genes, and TGF-β, TGFR-1, IGF-2 and IGFR-2 in CSFs all indicated that they may control the activation state of CSFs and regulate ECM remodeling. Our data also found that CSFs may be involved in the regulation of antitumor immune response through the secretion of CXCL12 and the interaction between CSFs and immune cells through MIF-CD74 pair. In addition, we found that high proportions of CSFs were associated with poor prognosis in BCa and LUAD, while total fibroblast proportions did not show significant association. Thus, we proposed that CSFs could be effective targets for cancer treatment.

To ensure specific targeting of CSFs, we also tried to identify the membrane molecules that were specifically expressed in CSFs. We found that FAP, LRRC15, ITGA11 and SPHK1 were specifically expressed in CSFs, especially LRRC15 and ITGA11 due to their more specific expression in CSFs. Current studies have shown that some of these molecules have been extensively applied in both preclinical and clinical studies. For example, FAP-targeted PET/CT or PET/MRI has shown diagnostic value in multiple cancers, such as lung cancer, breast cancer, HNSCC and gastric cancer (Backhaus et al., 2022; Kumar et al., 2022; Promteangtrong et al., 2022; Wang et al., 2022). In preclinical studies, the LRRC15-targeting antibody-drug conjugate ABBV-085 showed antitumor effect in breast cancer and ovarian cancer (Purcell et al., 2018; Ray et al., 2022). We believe that strategies targeting ITGA11 and SPHK1 could also have diagnostic and therapeutic value. Besides these membrane molecules, the cytokines that inhibit antitumor immune response or promote the transition of normal fibroblasts to CSFs, such as MIF, SPP1/OPN, could also be considered as therapeutic targets for cancer treatment (as proposed in Figure 7F).

This study also had limitations. First, we only analyzed limited scRNA-seq datasets, including ten datasets across eight tumor types and six datasets from five types of adjacent normal tissues. It is for sure that analysis of more scRNA-seq datasets will obtain more accurate results. Second, the data in this study only came from integrated analysis of scRNA-seq datasets, and the proposed features and functions of CSFs still need to be validated by experimental methods, such as mass cytometry, single-cell proteomics and multiplex staining techniques.

In summary, through comparison between fibroblasts in tumor tissues and normal tissues at single-cell level, we identified that COL11A1^+^ FBs specifically exist in tumor tissues, but not normal tissues, thus we named them CSFs. We further revealed that CSFs originate from normal fibroblasts. CSFs are in a more active state than other fibroblasts and may promote tumor progression through enhancing ECM remodeling and inhibiting antitumor immune response. We demonstrated that CSFs could be potential targets for cancer treatment, and membrane molecules FAP, LRRC15, ITGA11 and SPHK1 could be used as CSFs specific targets.

## Data Availability

The original contributions presented in the study are included in the article/Supplementary Material; further inquiries can be directed to the corresponding authors.
