# Supplementary material for: Single-cell analysis reveals the COL11A1+ fibroblasts are cancer-specific fibroblasts that promote tumor progression
Source: Front Pharmacol. 2023 Jan 20;14:1121586. doi: 10.3389/fphar.2023.1121586 (PMC9894880; doi:10.3389/fphar.2023.1121586)
Supplement: Supplementary file 10 [file DataSheet1.DOCX]

Supplementary Material

# Supplementary Figures and Tables

## Supplementary Figures





**Supplementary Figure 1.**

**The construction of multi-tumor cell atlas and cell atlas across normal tissues.**

**A**, UMAP depicting tumor origins of cell populations. **B**, tSNE visualization of the cell populations in tumor tissues from ten scRNA-seq datasets across eight tumor types. **C**, Marker gene expression for each cell type in multi-tumor, where dot size and color represent the percentage of marker gene expression (Pct. exp) and the averaged scaled expression (Avg. exp. scale) value, respectively. **D**, UMAP depicting tissue origins of cell populations. **E**, tSNE visualization of the cell populations from six scRNA-seq datasets of five types of normal tissues. **F**, Marker gene expression for each cell type across normal tissues, where dot size and color represent the percentage of marker gene expression (Pct. exp) and the averaged scaled expression (Avg. exp. scale) value, respectively.





**Supplementary Figure 2.**

**The construction of human fibroblast atlas across normal tissues and tumors.**

**A**, UMAP depicting the tissue origins of the fibroblast subtypes. **B**, UMAP depicting cell-cycle states of fibroblast subtypes. **C**, Violin plots showing marker genes for six fibroblast subtypes. **D**, Dot plots to show the expression of top 15 DEGs of CSFs in each cell type of normal tissues and tumors.


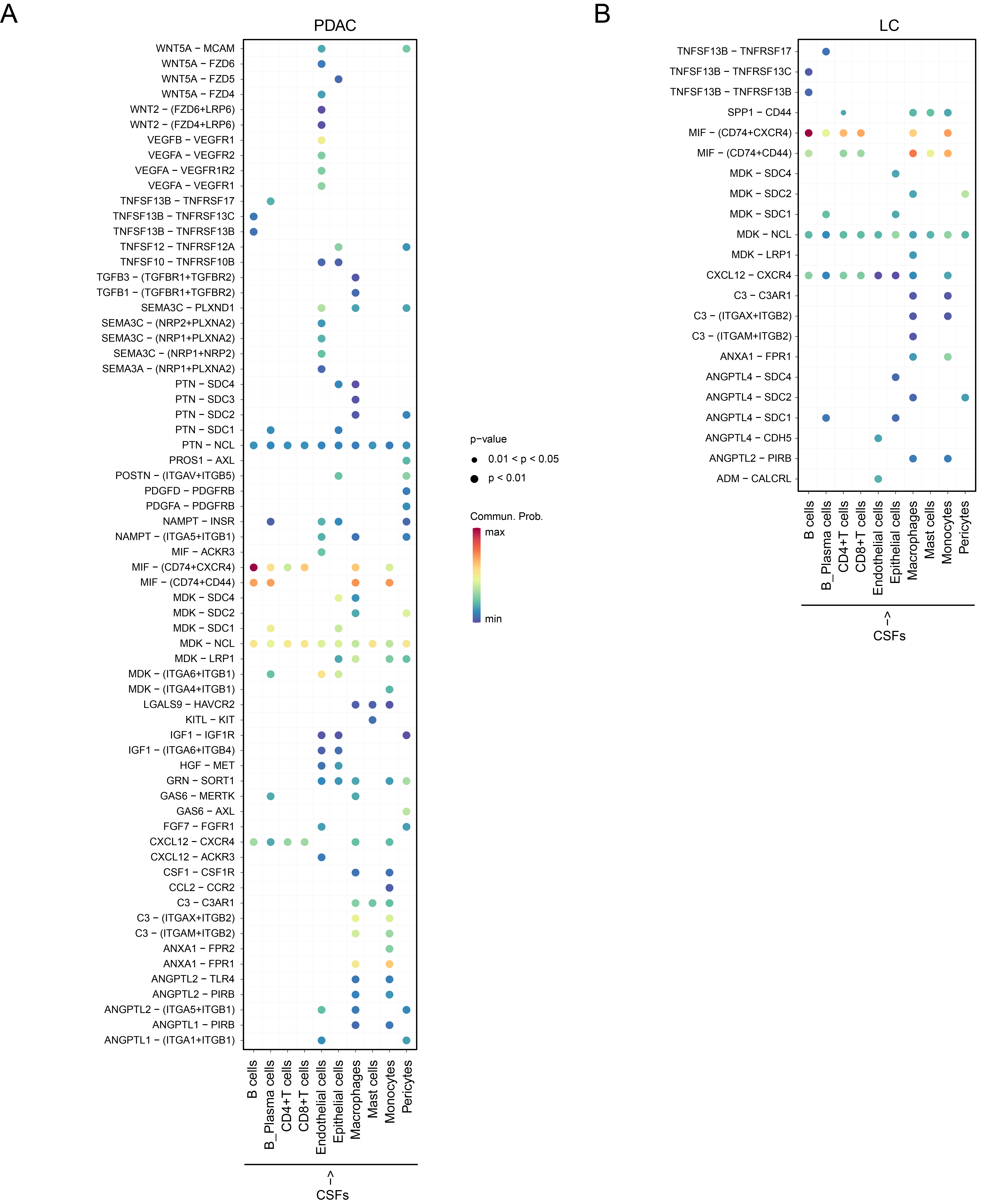


**Supplementary Figure 3.**

**Cell-cell communication analysis of CSFs and other cell types for cancers.** Interaction analysis showing enriched receptor-ligand pairs in CSFs and other cell types for PDAC (**A**) and LC (**B**).


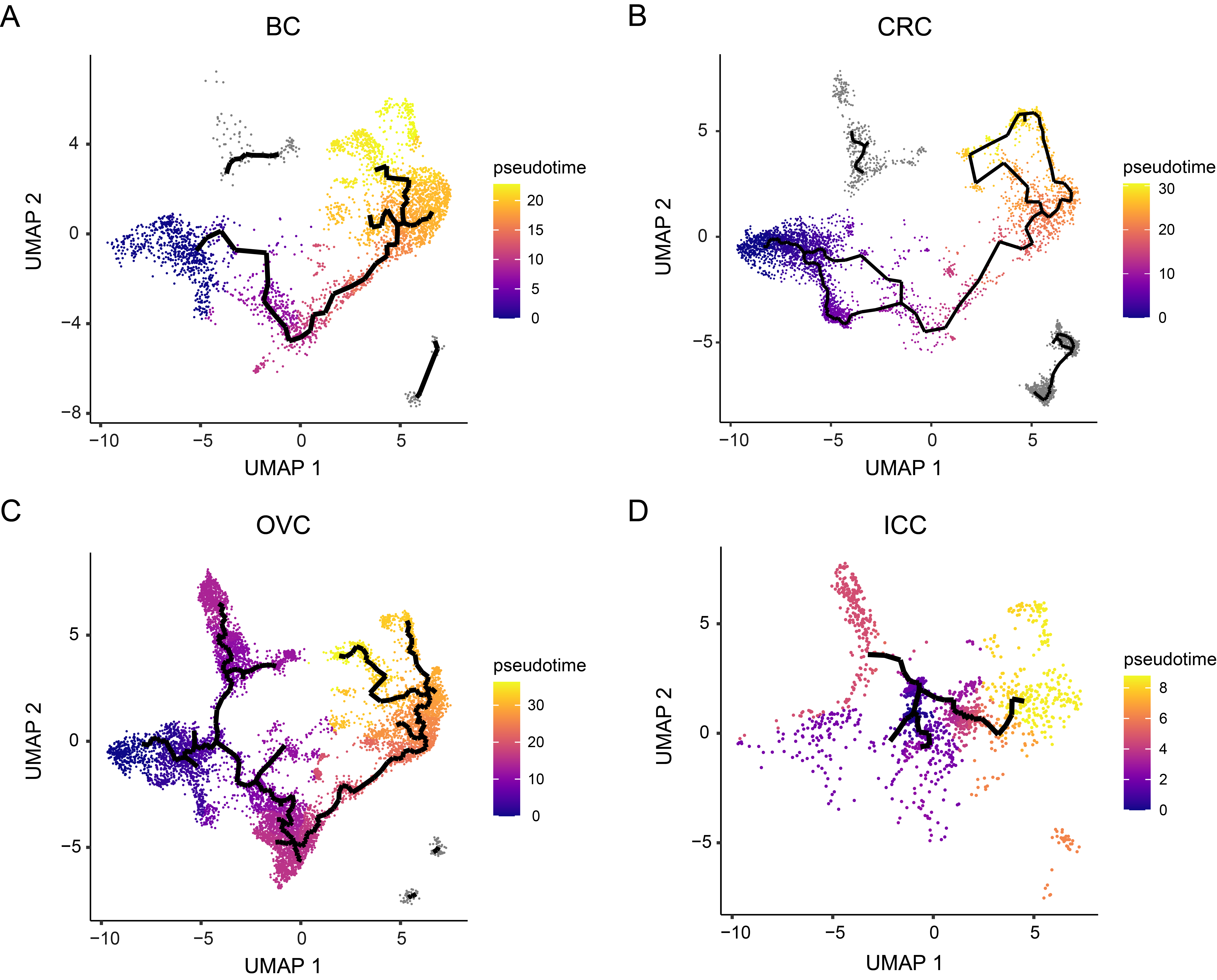


**Supplementary Figure 4.**

**Independent pseudotime analysis of fibroblast datasets from individual cancer.**

UMAP of fibroblasts to show the pseudotime trajectory in BC **(A)**, CRC **(B)**, OVC **(C)**, and ICC **(D)**. Pseudotime values code the cell color.


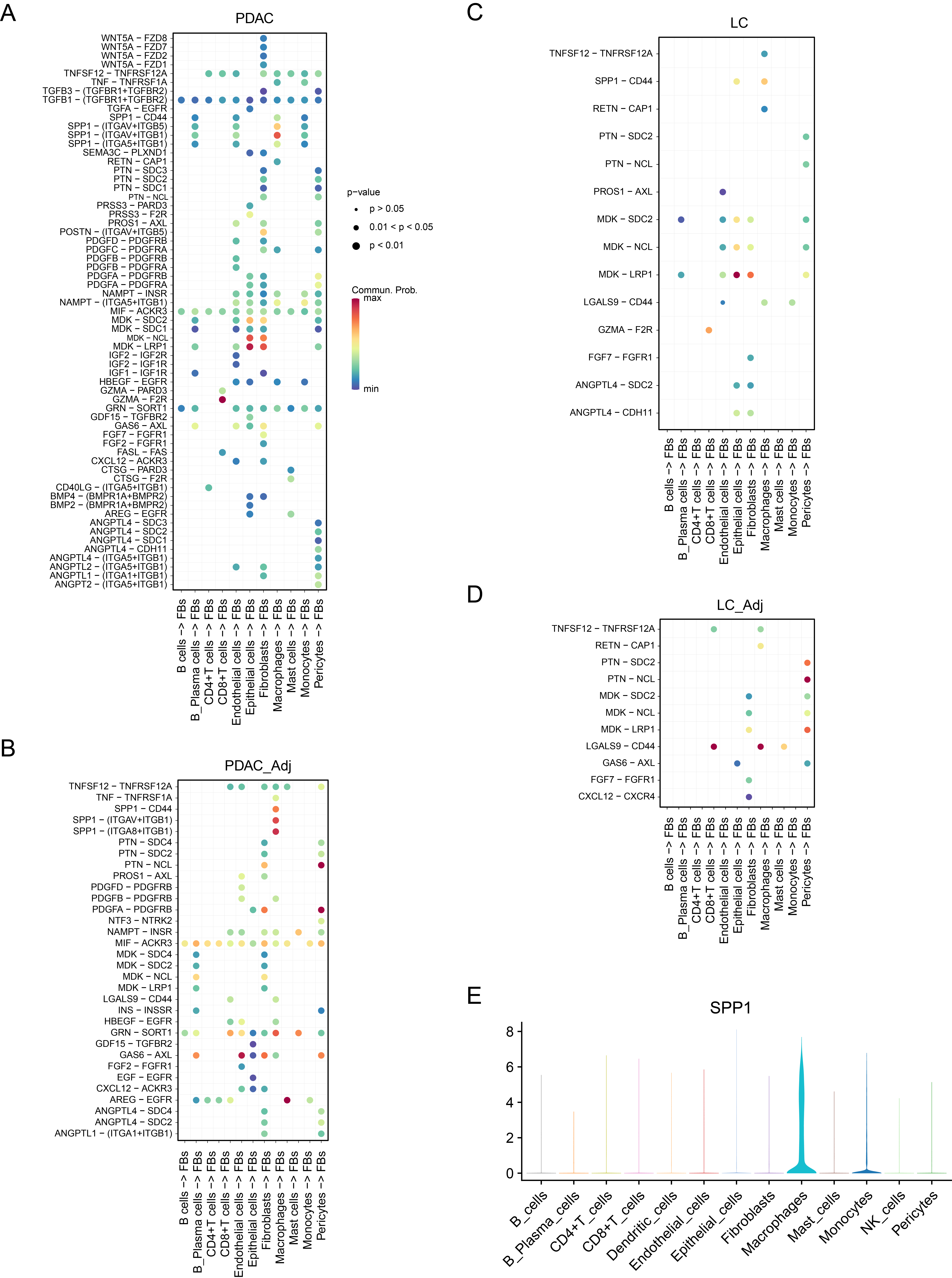


**Supplementary Figure 5.**

**Cell-cell communication analysis of fibroblasts and other cell types in tumor and normal tissues.**

Interaction analysis showing enriched receptor-ligand pairs in fibroblasts and other cell types for PDAC (**A**), normal pancreas tissues (**B**), LC (**C**), and normal lung tissues (**D**). **E**, Violin plots showing the expression of SPP1 for cell populations.


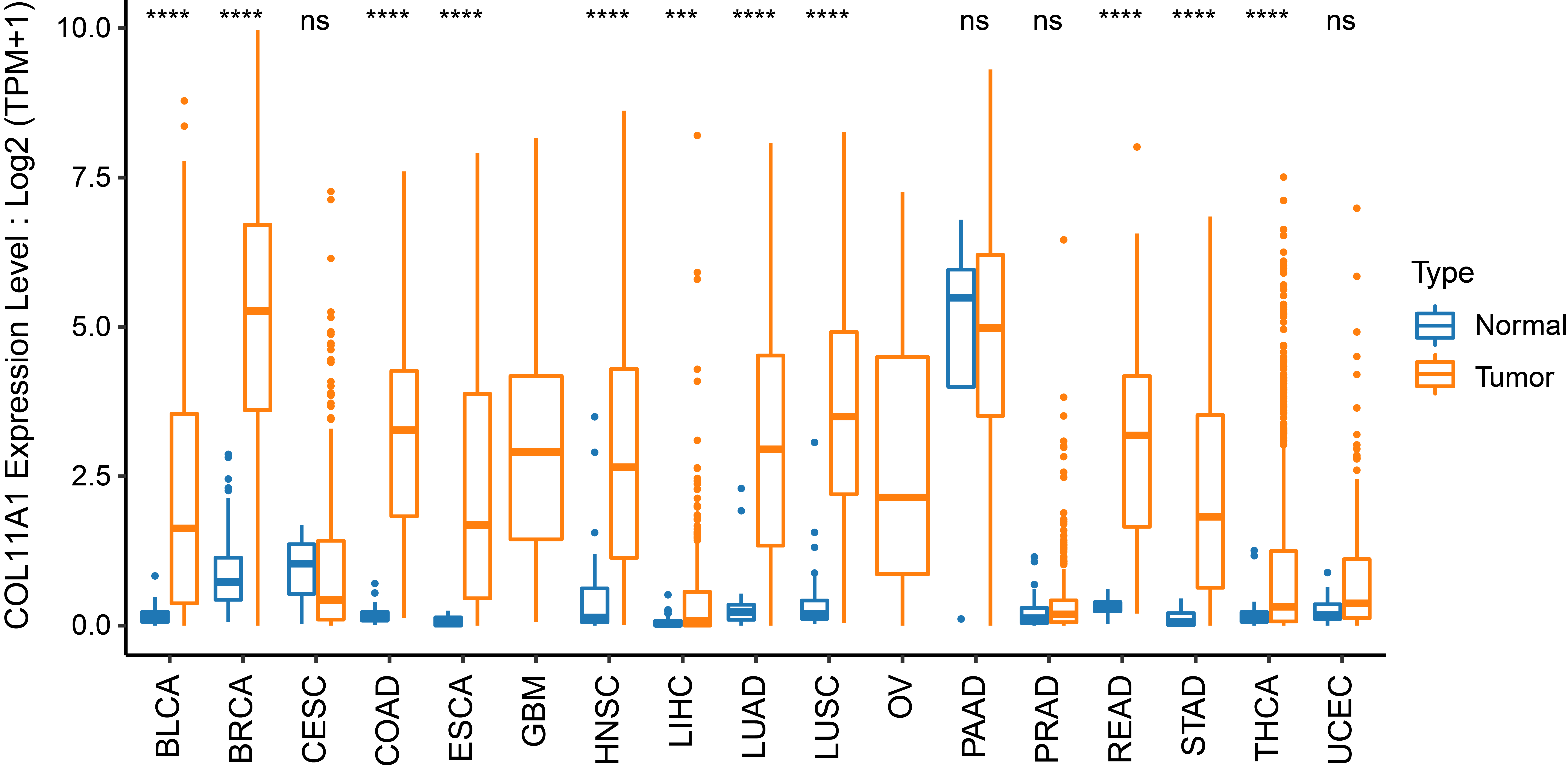


**Supplementary Figure 6.**

**The expression differences of COL11A1 between normal and tumor tissues from the TCGA database.** ns, not significant; *p < 0.05; **p < 0.01; ***p < 0.001; ****p < 0.0001.


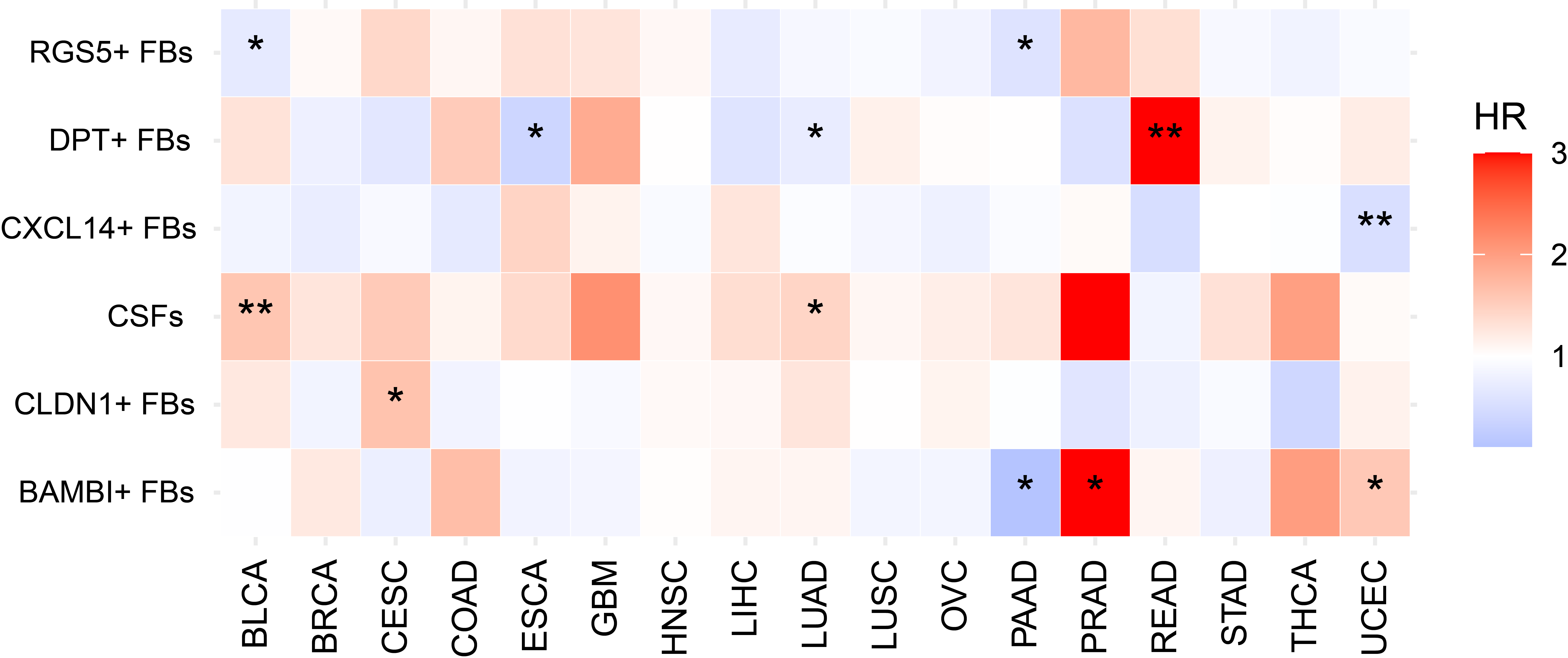


**Supplementary Figure 7.**

**The clinical relevance of fibroblast subtypes for multi-cancer.**

Heatmap showing the HRs and statistical significance for 6 fibroblast subtypes, where color represent HR value [red, HR above 1 (poor prognosis); and blue, HR below 1 (favorable prognosis)]. *p < 0.05, **p < 0.01. HR, hazard ratio.


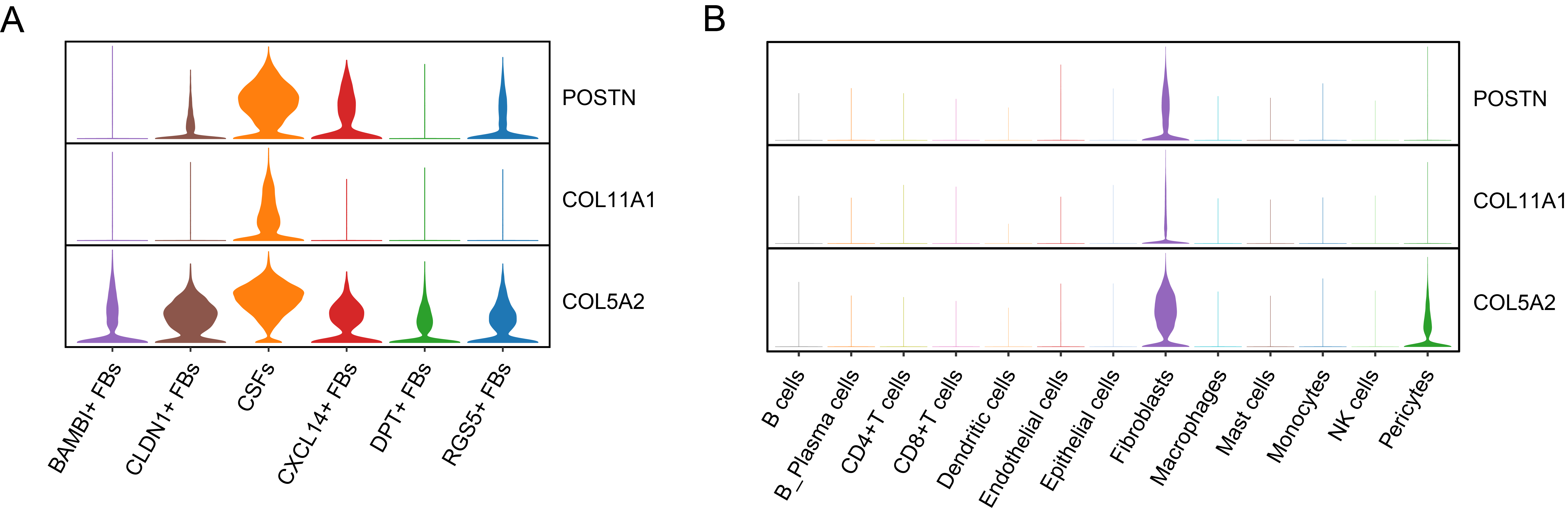


**Supplementary Figure 8.**

**POSTN, COL11A1, and COL5A2 are highly expressed in CSFs.**

**A** and **B**, Violin plots to show the expression levels of the three highly expressed genes in CSFs (POSTN, COL11A1, and COL5A2) in different fibroblast clusters (A) and different cell types (B).

## Supplementary Tables

**Supplementary Table 1:** The information of integrated scRNA-seq datasets.

**Supplementary Table 2:** The number of cells from each tissue.

**Supplementary Table 3:** Top 50 differentially expressed genes of each cell population for multi-cancer.

**Supplementary Table 4:** Top 50 differentially expressed genes of each cell population across normal tissues.

**Supplementary Table 5:** Top 50 differentially expressed genes of each fibroblast subtype across normal tissues and tumors.

**Supplementary Table 6:** Cell numbers of COL11A1^+^ FBs from each tissue origin.

**Supplementary Table 7:** Proportions of fibroblast subtypes in total fibroblasts from each tissue type.

**Supplementary Table 8:** Signature gene matrix based on single-cell RNA sequencing dataset of multi-cancer.

**Supplementary Table 9:** Baseline characteristics of enrolled bladder cancer patients.
